# Supplementary material for: Longitudinal profiles of plasma gelsolin, cytokines and antibody expression predict COVID-19 severity and hospitalization outcomes
Source: Front Immunol. 2022 Sep 6;13:1011084. doi: 10.3389/fimmu.2022.1011084 (PMC9489255; doi:10.3389/fimmu.2022.1011084)
Supplement: Supplementary file 1 [file Presentation_1.pptx]

## Slide 1
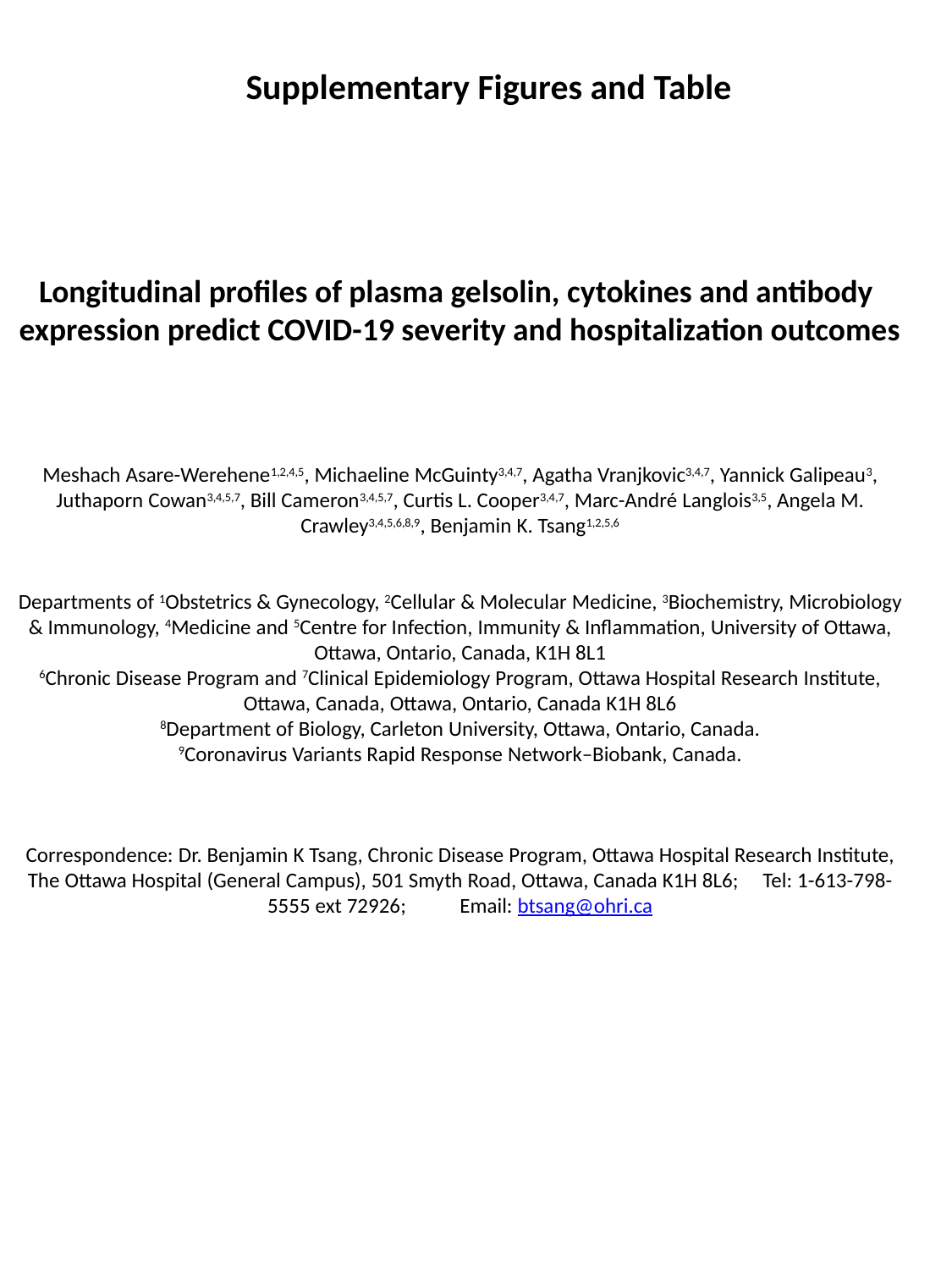

Supplementary Figures and Table
Longitudinal profiles of plasma gelsolin, cytokines and antibody
expression predict COVID-19 severity and hospitalization outcomes
Meshach Asare-Werehene1,2,4,5, Michaeline McGuinty3,4,7, Agatha Vranjkovic3,4,7, Yannick Galipeau3, Juthaporn Cowan3,4,5,7, Bill Cameron3,4,5,7, Curtis L. Cooper3,4,7, Marc-André Langlois3,5, Angela M. Crawley3,4,5,6,8,9, Benjamin K. Tsang1,2,5,6
Departments of 1Obstetrics & Gynecology, 2Cellular & Molecular Medicine, 3Biochemistry, Microbiology & Immunology, 4Medicine and 5Centre for Infection, Immunity & Inflammation, University of Ottawa, Ottawa, Ontario, Canada, K1H 8L1
6Chronic Disease Program and 7Clinical Epidemiology Program, Ottawa Hospital Research Institute, Ottawa, Canada, Ottawa, Ontario, Canada K1H 8L6
8Department of Biology, Carleton University, Ottawa, Ontario, Canada.
9Coronavirus Variants Rapid Response Network–Biobank, Canada.
Correspondence: Dr. Benjamin K Tsang, Chronic Disease Program, Ottawa Hospital Research Institute, The Ottawa Hospital (General Campus), 501 Smyth Road, Ottawa, Canada K1H 8L6; Tel: 1-613-798-5555 ext 72926; Email: btsang@ohri.ca

## Slide 2
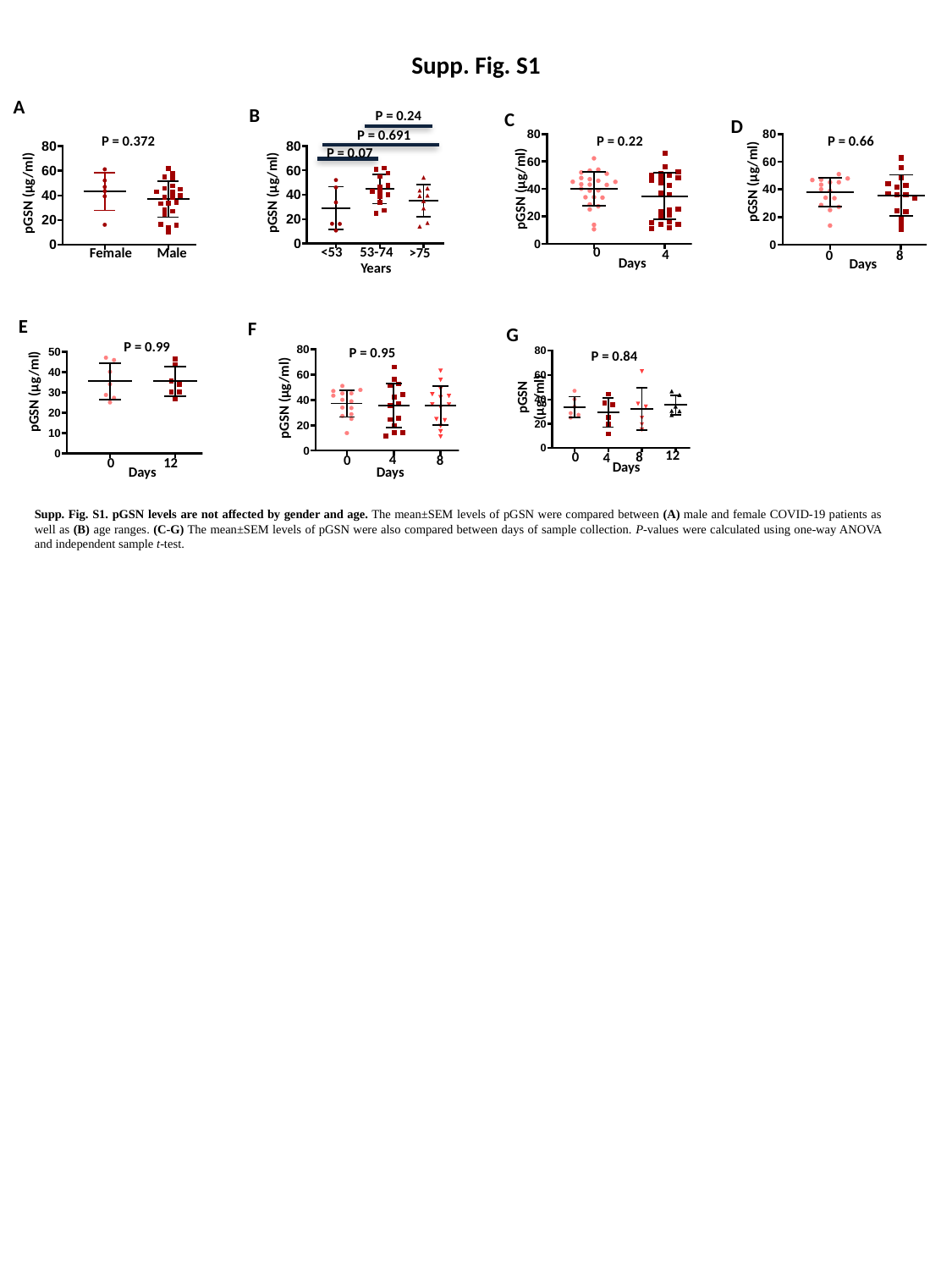

Supp. Fig. S1
A
B
P = 0.24
C
D
P = 0.691
pGSN (μg/ml)
0
8
Days
P = 0.66
P = 0.372
P = 0.22
pGSN (μg/ml)
0
4
Days
P = 0.07
pGSN (µg/ml)
pGSN (µg/ml)
<53
53-74
Female
Male
>75
Years
E
F
G
pGSN (μg/ml)
0
12
Days
P = 0.99
pGSN (μg/ml)
4
0
8
Days
P = 0.95
P = 0.84
pGSN (μg/ml)
12
8
0
4
Days
Supp. Fig. S1. pGSN levels are not affected by gender and age. The mean±SEM levels of pGSN were compared between (A) male and female COVID-19 patients as well as (B) age ranges. (C-G) The mean±SEM levels of pGSN were also compared between days of sample collection. P-values were calculated using one-way ANOVA and independent sample t-test.

## Slide 3
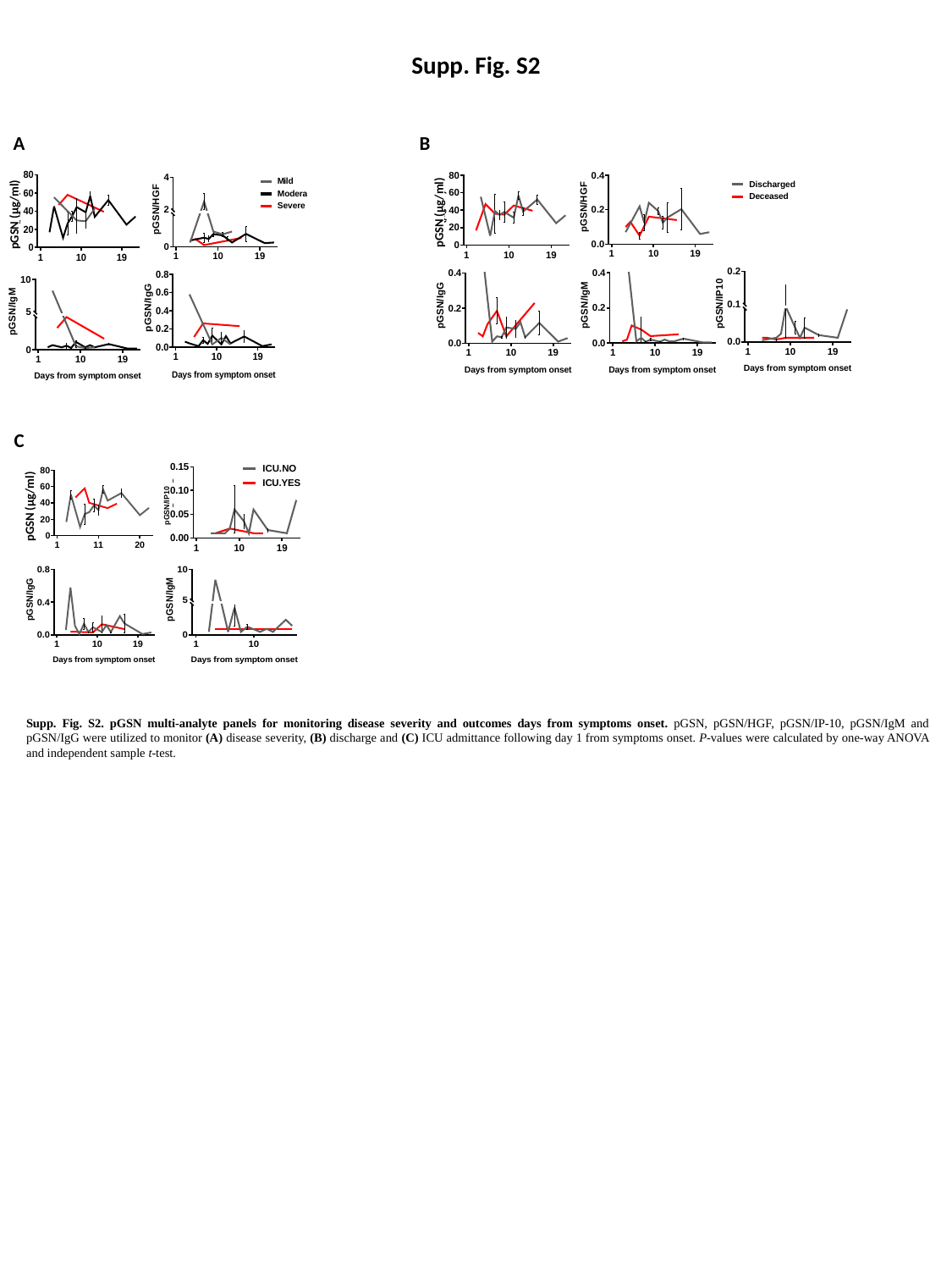

# Supp. Fig. S2
A
B
pGSN (μg/ml)
pGSN (μg/ml)
C
pGSN (μg/ml)
Supp. Fig. S2. pGSN multi-analyte panels for monitoring disease severity and outcomes days from symptoms onset. pGSN, pGSN/HGF, pGSN/IP-10, pGSN/IgM and pGSN/IgG were utilized to monitor (A) disease severity, (B) discharge and (C) ICU admittance following day 1 from symptoms onset. P-values were calculated by one-way ANOVA and independent sample t-test.

## Slide 4
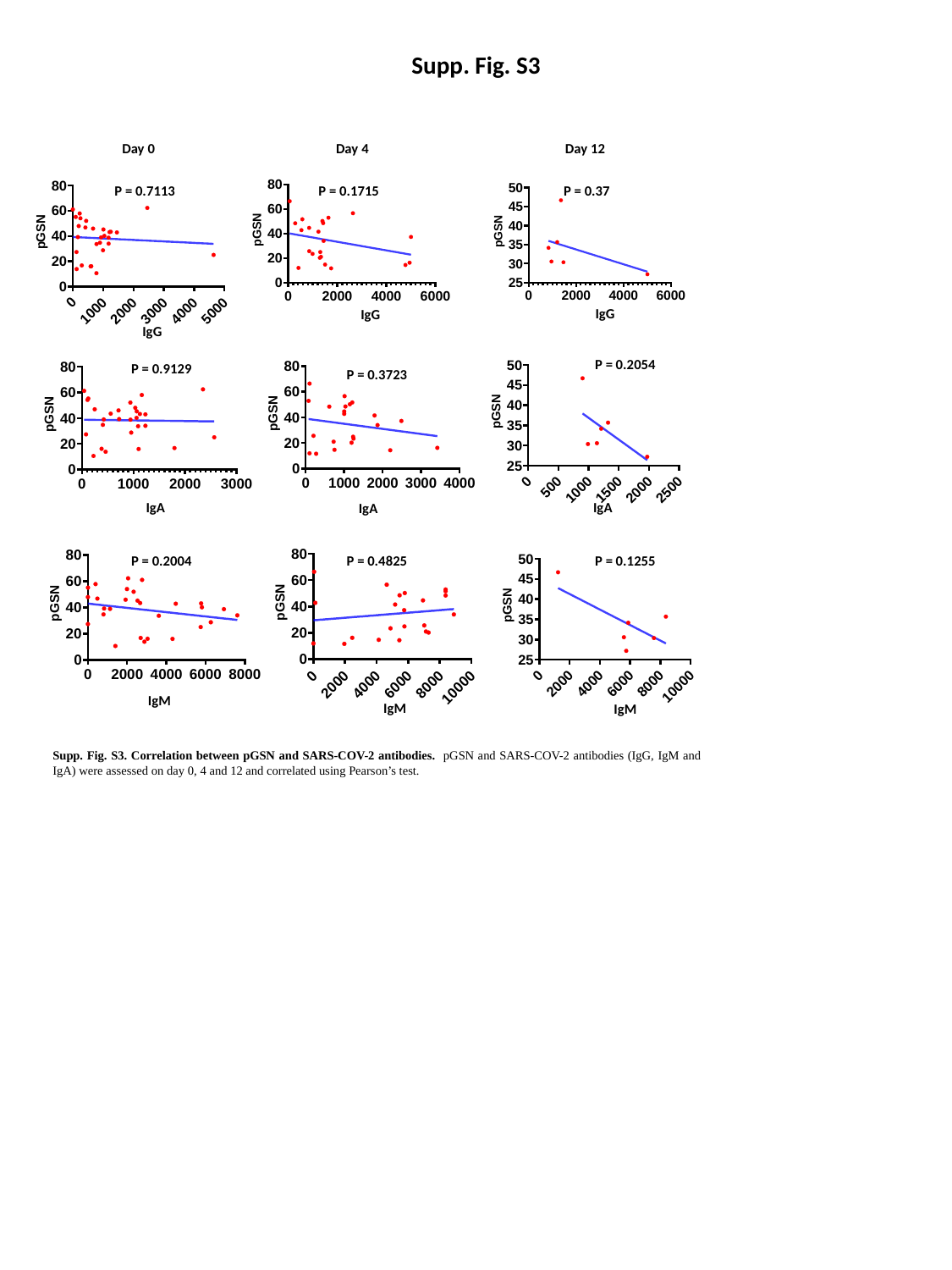

# Supp. Fig. S3
Day 4
Day 0
Day 12
P = 0.7113
IgG
P = 0.1715
IgG
P = 0.37
IgG
P = 0.2054
IgA
P = 0.9129
IgA
P = 0.3723
IgA
P = 0.2004
IgM
P = 0.4825
IgM
P = 0.1255
IgM
Supp. Fig. S3. Correlation between pGSN and SARS-COV-2 antibodies. pGSN and SARS-COV-2 antibodies (IgG, IgM and IgA) were assessed on day 0, 4 and 12 and correlated using Pearson’s test.

## Slide 5
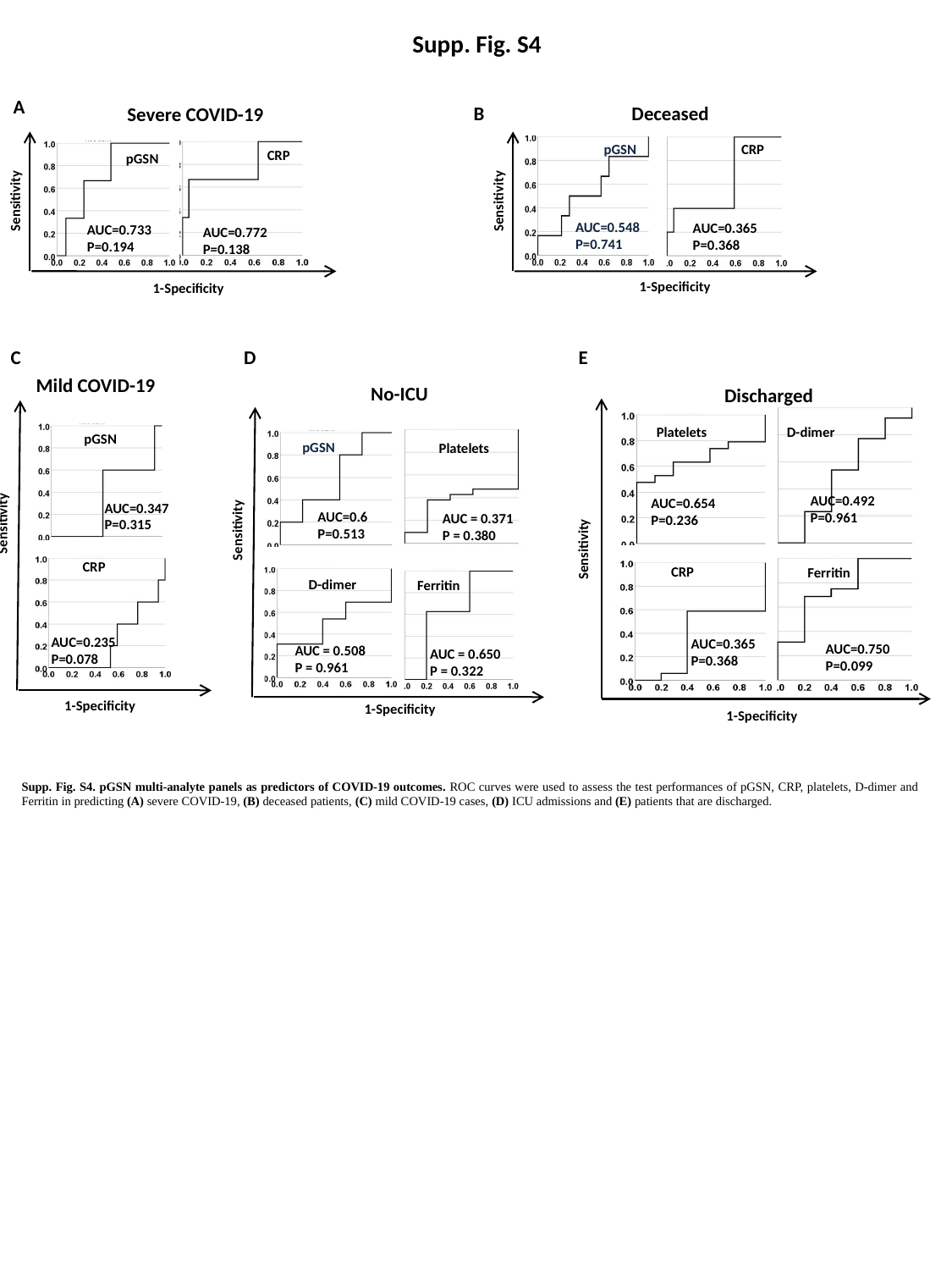

# Supp. Fig. S4
A
B
Deceased
Severe COVID-19
pGSN
CRP
CRP
pGSN
Sensitivity
Sensitivity
AUC=0.548
P=0.741
AUC=0.365
P=0.368
AUC=0.733
P=0.194
AUC=0.772
P=0.138
1-Specificity
1-Specificity
C
D
E
Mild COVID-19
No-ICU
Discharged
Platelets
D-dimer
pGSN
pGSN
Platelets
AUC=0.492
P=0.961
AUC=0.654
P=0.236
AUC=0.347
P=0.315
AUC=0.6
P=0.513
AUC = 0.371
P = 0.380
Sensitivity
Sensitivity
Sensitivity
CRP
AUC=0.235
P=0.078
CRP
Ferritin
D-dimer
Ferritin
AUC=0.365
P=0.368
AUC=0.750
P=0.099
AUC = 0.508
P = 0.961
AUC = 0.650
P = 0.322
1-Specificity
1-Specificity
1-Specificity
Supp. Fig. S4. pGSN multi-analyte panels as predictors of COVID-19 outcomes. ROC curves were used to assess the test performances of pGSN, CRP, platelets, D-dimer and Ferritin in predicting (A) severe COVID-19, (B) deceased patients, (C) mild COVID-19 cases, (D) ICU admissions and (E) patients that are discharged.

## Slide 6
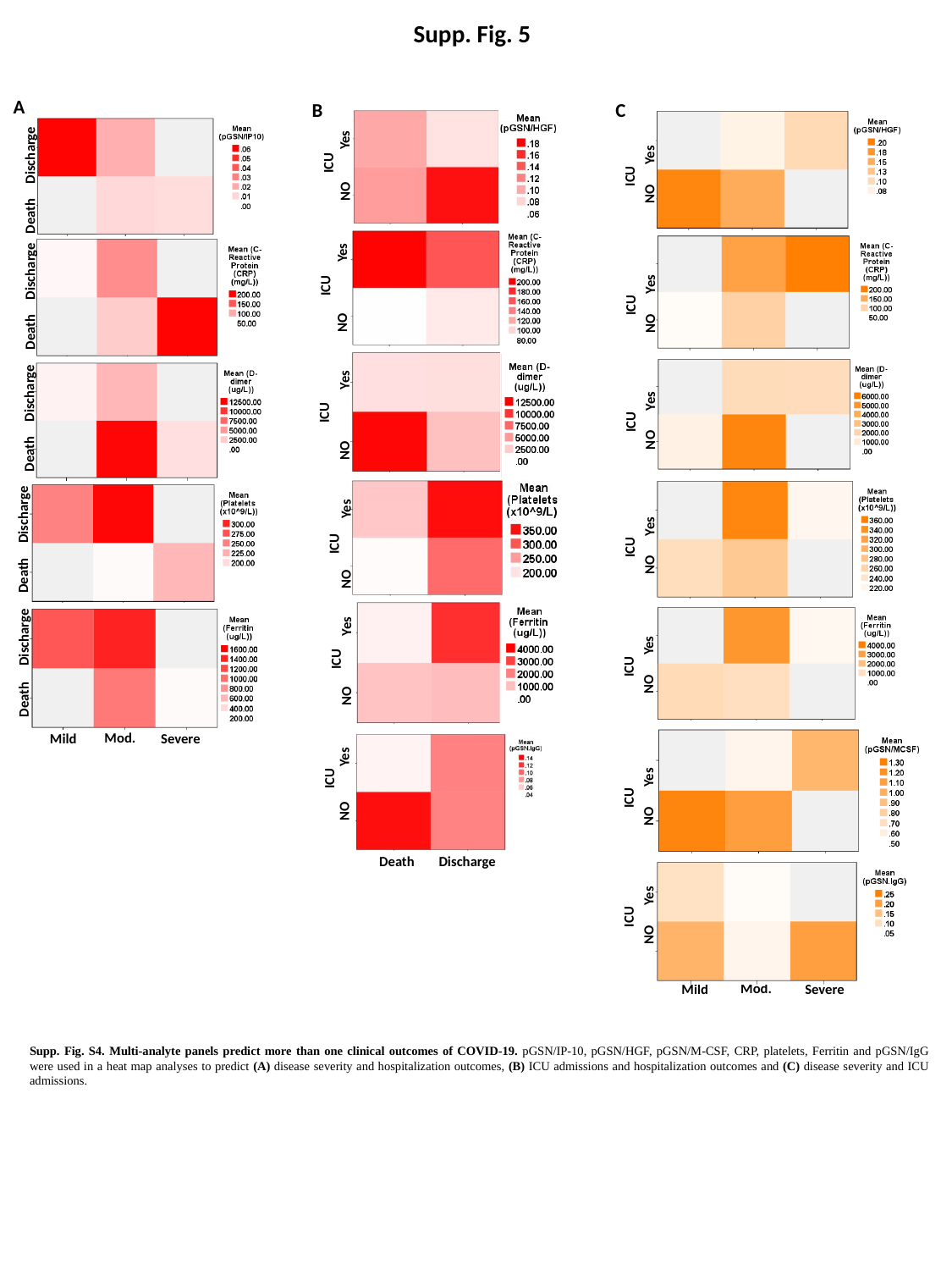

# Supp. Fig. 5
A
B
C
Yes
Yes
Discharge
ICU
ICU
NO
NO
Death
Yes
Discharge
Yes
ICU
ICU
NO
NO
Death
Yes
Discharge
Yes
ICU
ICU
NO
NO
Death
Yes
Discharge
Yes
ICU
ICU
NO
Death
NO
Yes
Discharge
Yes
ICU
ICU
NO
NO
Death
Mod.
Severe
Mild
Yes
Yes
ICU
ICU
NO
NO
Death
Discharge
Yes
ICU
NO
Mod.
Severe
Mild
Supp. Fig. S4. Multi-analyte panels predict more than one clinical outcomes of COVID-19. pGSN/IP-10, pGSN/HGF, pGSN/M-CSF, CRP, platelets, Ferritin and pGSN/IgG were used in a heat map analyses to predict (A) disease severity and hospitalization outcomes, (B) ICU admissions and hospitalization outcomes and (C) disease severity and ICU admissions.

## Slide 7
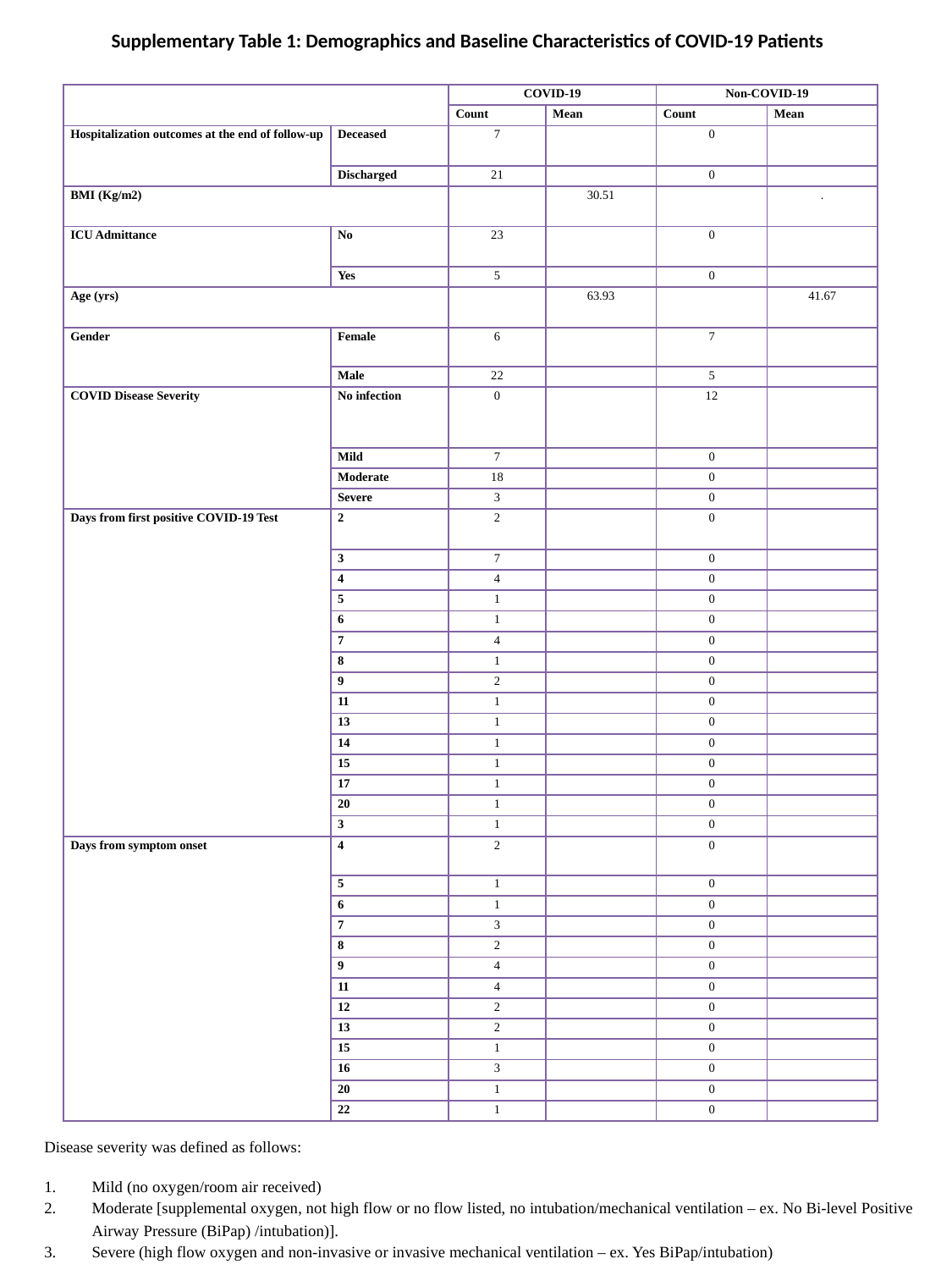

# Supplementary Table 1: Demographics and Baseline Characteristics of COVID-19 Patients
| | | COVID-19 | | Non-COVID-19 | |
| --- | --- | --- | --- | --- | --- |
| | | Count | Mean | Count | Mean |
| Hospitalization outcomes at the end of follow-up | Deceased | 7 | | 0 | |
| | Discharged | 21 | | 0 | |
| BMI (Kg/m2) | | | 30.51 | | . |
| ICU Admittance | No | 23 | | 0 | |
| | Yes | 5 | | 0 | |
| Age (yrs) | | | 63.93 | | 41.67 |
| Gender | Female | 6 | | 7 | |
| | Male | 22 | | 5 | |
| COVID Disease Severity | No infection | 0 | | 12 | |
| | Mild | 7 | | 0 | |
| | Moderate | 18 | | 0 | |
| | Severe | 3 | | 0 | |
| Days from first positive COVID-19 Test | 2 | 2 | | 0 | |
| | 3 | 7 | | 0 | |
| | 4 | 4 | | 0 | |
| | 5 | 1 | | 0 | |
| | 6 | 1 | | 0 | |
| | 7 | 4 | | 0 | |
| | 8 | 1 | | 0 | |
| | 9 | 2 | | 0 | |
| | 11 | 1 | | 0 | |
| | 13 | 1 | | 0 | |
| | 14 | 1 | | 0 | |
| | 15 | 1 | | 0 | |
| | 17 | 1 | | 0 | |
| | 20 | 1 | | 0 | |
| | 3 | 1 | | 0 | |
| Days from symptom onset | 4 | 2 | | 0 | |
| | 5 | 1 | | 0 | |
| | 6 | 1 | | 0 | |
| | 7 | 3 | | 0 | |
| | 8 | 2 | | 0 | |
| | 9 | 4 | | 0 | |
| | 11 | 4 | | 0 | |
| | 12 | 2 | | 0 | |
| | 13 | 2 | | 0 | |
| | 15 | 1 | | 0 | |
| | 16 | 3 | | 0 | |
| | 20 | 1 | | 0 | |
| | 22 | 1 | | 0 | |
Disease severity was defined as follows:
Mild (no oxygen/room air received)
Moderate [supplemental oxygen, not high flow or no flow listed, no intubation/mechanical ventilation – ex. No Bi-level Positive Airway Pressure (BiPap) /intubation)].
Severe (high flow oxygen and non-invasive or invasive mechanical ventilation – ex. Yes BiPap/intubation)
